# Complete Genome Sequence of *Flavobacterium* sp. Strain GSB-24, Isolated from inside *Dendrobium* Roots

**DOI:** 10.1128/mra.01343-22

**Published:** 2023-02-28

**Authors:** Tomoki Nishioka, Kana Morinaga, Hideyuki Tamaki

**Affiliations:** a Bioproduction Research Institute, National Institute of Advanced and Industrial Science and Technology (AIST), Tsukuba, Ibaraki, Japan; b Faculty of Life and Environmental Sciences, University of Tsukuba, Tsukuba, Ibaraki, Japan; c Microbiology Research Center for Sustainability (MiCS), University of Tsukuba, Tsukuba, Ibaraki, Japan; d Biotechnology Research Center, The University of Tokyo, Tokyo, Japan; University of Arizona

## Abstract

Here, we report a complete genome sequence of *Flavobacterium* sp. strain GSB-24, an endophytic bacterium of *Dendrobium* plants. The genome consists of a circular 5,286,830-bp chromosome with a G+C content of 33.8% and a circular 64,374-bp plasmid with a G+C content of 29.3%.

## ANNOUNCEMENT

Members of the genus *Flavobacterium* ubiquitously inhabit various plant species and have been thought to play an important role in promoting plant growth through protection from plant diseases, solubilization of phosphate, and production of indole-3-acetic acid and 1-aminocyclopropane-1-carboxylate deaminase (1–4). However, little is known about the role of *Flavobacterium* species in the growth of *Dendrobium* plants. We recently isolated various endophytic bacteria of green stem-*Dendrobium* roots using diluted R2A media prepared by autoclaving phosphate and agar separately (PS-DR2A) (5). Based on the 16S rRNA gene sequencing analysis (5), one of the isolates, designated GSB-24, was classified into genus *Flavobacterium*. Here, we provide the complete genome sequence of *Flavobacterium* sp. strain GSB-24.

The single colony of strain GSB-24 was cultured in 1/5 strength tryptic soy broth (Becton, Dickinson and Company, Sparks, MD) at 25°C with shaking at 120 rpm for 24 h. The cultured cells were lysed with lysozyme and achromopeptidase (6), and then genomic DNA was extracted using the phenol-chloroform method as described previously (7). The obtained genomic DNA was subjected to the following sequencing analyses. The MiSeq sequencing platform (Illumina, San Diego, CA) and GridION X5 system (Oxford Nanopore Technologies [ONT], Oxford, UK) were used to conduct *de novo* sequencing. For MiSeq sequencing, we used the Nextera DNA flex library prep kit (Illumina) to construct a sequencing library and obtained paired-end (2 × 256 bp) reads. Default parameters were used for all software unless otherwise specified. The raw sequencing data (883,424 reads; average read length, 248.1 bp) were processed using fastp v.0.20.1 (8) to trim adapters and select high-quality data (Phred quality score, >30), which resulted in 835,176 reads with an average length of 242.5 bp. For GridION X5 sequencing, a sequencing library was prepared using a ligation sequencing kit (SQK-LSK110; ONT) without shearing. The constructed library was applied to an R9.4.1 flow cell (FLO-MIN106; ONT). GridION X5 sequencing yielded a total of 752,860 reads with an *N*_50_ value of 5,169 bp. A quality control step using Filtlong v.0.2.1 (https://github.com/rrwick/Filtlong) selected sequence reads that were >3,000 bp long, with a >90% identity match to the Illumina short reads, which generated a total of 87,504 reads (800 Mb) with an *N*_50_ value of 11,962 bp.

For complete *de novo* genome assembly, the processed short and long reads were coassembled using the Unicycler v.0.4.8 pipeline (9). The presence of contaminant DNA was assessed using BlobTools v.1.0 (10). The contig sequence obtained was polished using Pilon v.1.24 (11). CheckM v.1.2.2 was used to check the quality of the assembled genome sequence, with a completeness of 99.7% and contamination of 0.6% (12). Afterward, we performed automatic annotation using the annotation pipeline DFAST v.1.2.18 (13). The complete *Flavobacterium* sp. strain GSB-24 genome comprises a circular 5,286,830-bp chromosome with a G+C content of 33.8%, a circular 64,374-bp plasmid with a G+C content of 29.3%, 4,459 coding sequences, 18 rRNA genes, and 64 tRNA genes.

### Data availability.

The complete genome sequence and annotations of *Flavobacterium* sp. strain GSB-24 have been deposited at DDBJ/EMBL/GenBank under accession numbers AP027043 and AP027044. The genome sequence also has been submitted to the SRA under BioSample number SAMD00557613 and BioProject number PRJDB14765. Raw sequence data for strain GSB-24 were deposited under DRA accession numbers DRR420785 (Illumina) and DRR420786 (Nanopore).
